# Impact of *DARC* rs12075 Variants on Liver Fibrosis Progression in Patients with Chronic Hepatitis C: A Retrospective Study

**DOI:** 10.3390/biom9040143

**Published:** 2019-04-09

**Authors:** María Ángeles Jiménez-Sousa, Ana Zaida Gómez-Moreno, Daniel Pineda-Tenor, Juan José Sánchez-Ruano, Tomas Artaza-Varasa, María Martin-Vicente, Amanda Fernández-Rodríguez, Isidoro Martínez, Salvador Resino

**Affiliations:** 1 Unidad de Infección Viral e Inmunidad, Centro Nacional de Microbiología, Instituto de Salud Carlos III, 28220 Majadahonda, Spain; jimenezsousa@isciii.es (M.A.J.-S.); maria.martinv@externos.isciii.es (M.M.-V.); amandafr@isciii.es (A.F.-R.); imago@isciii.es (I.M.); sresino@isciii.es (S.R.); 2 Servicio de Digestivo, Hospital Virgen de la Salud, 45004 Toledo, Spain; ana.zaidag@hotmail.com (A.Z.G.-M.); jjsanchezr@sescam.jccm.es (J.J.S.-R.); tdeartaza@gmail.com (T.A.-V.); 3 Servicio de Laboratorio Clínico, Hospital de Antequera, 29200 Málaga, Spain; dpinedatenor@gmail.com (D.P.-T.)

**Keywords:** chronic hepatitis C, liver stiffness, hepatic fibrosis, cirrhosis, *DARC*, single nucleotide polymorphisms

## Abstract

The Duffy antigen receptor for chemokines (*DARC*) rs12075 polymorphism regulates leukocyte trafficking and proinflammatory chemokine homeostasis. Hepatitis C virus (HCV)-mediated liver fibrosis is associated with an uncontrolled inflammatory response. In this study, we evaluate the association between the *DARC* rs12075 polymorphism and liver stiffness progression in HCV-infected patients. We carried out a retrospective cohort study (repeated measures design) in 208 noncirrhotic patients with chronic hepatitis C (CHC) who had at least two liver stiffness measurements (LSM) with a separation of at least 12 months. We used generalized linear models to analyze the association between *DARC* rs12075 polymorphism and outcome variables. During a follow-up of 46.6 months, the percentage of patients with stages of fibrosis F0/F1 decreased (*p* < 0.001), while LSM values and the percentage of patients with cirrhosis increased (*p* < 0.001). This pattern of changes was maintained in each of the groups of patients analyzed according to their rs12075 genotypes (AA or AG/GG). However, the variations in liver stiffness characteristics were lower in patients with the rs12075 AG/GG genotype (AG/GG versus AA). Thereby, in the adjusted analysis, patients with the rs12075 AG/GG genotype had a lower risk of an increased value of LSM2/LSM1 arithmetic mean ratio (AMR = 0.83; *p* = 0.001) and of an increase in LSM ≥ 5 kPa (odds ratio (OR) = 0.28; *p* = 0.009). Besides, patients with rs12075 AG/GG had a lower risk of cirrhosis progression (OR = 0.24; *p* = 0.009). No significant associations were found for an increase in LSM ≥ 10 kPa. We found an association between the *DARC* rs12075 single nucleotide polymorphism (SNP) and CHC progression. Specifically, patients with the *DARC* rs12075 AG/GG genotype had a lower risk of liver fibrosis progression and development of cirrhosis.

## 1. Introduction

Chronic hepatitis C (CHC) is a clinically relevant human infectious disease with a significant impact on the health system. The World Health Organization estimated that 71 million people are chronically infected with hepatitis C virus (HCV) [1]. These individuals frequently develop serious hepatic complications such as cirrhosis and hepatocellular carcinoma [2,3], even after successful treatment with direct-acting antivirals (DAAs), particularly patients with advanced fibrosis and cirrhosis [4,5]. This evolution depends, among other factors, on the individual’s genetic background, as several single nucleotide polymorphisms (SNPs) have been associated with liver disease progression [6,7].

Staging liver fibrosis is crucial for the management and prognosis of CHC patients [8], as early detection of fibrosis allows the implementation of preventive strategies that have a positive impact on the patient outcome [9,10]. Currently, the severity of liver fibrosis is mostly evaluated by noninvasive elastography techniques, particularly the evaluation of liver stiffness measurement (LSM), which gives us quantitative data about liver stiffness [11,12].

The development of advanced liver diseases is associated with an uncontrolled inflammation, characterized by the expression of high levels of cytokines and chemokines and recruitment of various leukocyte subsets into the liver [13,14]. In agreement with this, several studies have shown that SNPs in chemokine and chemokine receptor genes affect inflammation and fibrosis outcome in HCV infection [15,16]. Therefore, the analysis of the impact of individual SNPs on the development of liver cirrhosis may provide information about CHC pathogenesis and may be essential for taking preventive measures [10].

The Duffy antigen receptor for chemokines (DARC), also known as atypical chemokine receptor-1 or ACKR1, is a member of the chemokine decoy receptor (CDR) family, atypical chemokine binders capable of internalizing their cognate chemokine ligands [17]. Therefore, the CDRs regulate the action of primary proinflammatory cytokines and chemokines. The DARC belongs to the blood group system [18] and it is a receptor for various proinflammatory chemokines such as interleukin (IL)-8, monocyte chemoattractant protein-1 (MCP-1/CCL2), regulated on activation normal T-cell expressed and secreted (RANTES/CCL5), eotaxin-1 (CCL11), and thymus- and activation-regulated chemokine (TARC/CCL17) [19]. In addition to erythrocytes, DARC is expressed in other cell types, including endothelial cells [20]. On the endothelium, DARC seems to be implicated in the transcytosis of chemokines and presentation to blood leukocytes [21,22]. In contrast, in erythrocytes, DARC was suggested to promote the capture of plasma chemokines and their intracellular storage [23,24,25], avoiding the activation of leukocytes [21,22]. Because *DARC* rs12075 SNP (125A > G; Asp42Gly) is a major determinant of circulating CCL2 levels [26] and CCL2 has been associated with liver fibrosis progression [27], a relationship between rs12075 and liver cirrhosis is expected.

Our objective was to explore the association between the *DARC* rs12075 SNP and liver fibrosis progression, evaluated by LSM, in patients infected with HCV.

## 2. Materials and Methods

### 2.1. Study Population

We performed a retrospective cohort study (repeated measures design) in 208 patients with chronic hepatitis C from Hospital Virgen de la Salud (Toledo, Spain), recruited between 2008 and 2016. This work was conducted following the 1975 Declaration of Helsinki. The Institutional Review Board of the Instituto de Salud Carlos III (“Comités de Ética de la Investigación y Bienestar Animal”—4 April 2013) approved the study, and all patients signed the consent form.

The selection criteria were: (1) demonstrable plasma HCV RNA during the follow-up; (2) a DNA sample; (3) a baseline LSM (LSM1) and other final LSM (LSM2) with a separation of at least 12 months in medical history. The exclusion criteria in this study were: (1) baseline cirrhosis (F4; LSM1 ≥ 12.5 kPa); (2) coinfection with human immunodeficiency virus or hepatitis B virus; (3) autoimmune liver disease. Hepatitis C virus therapy could be administered before or after the baseline, but we only included nonresponder patients in this study (patients treated for HCV infection before starting the study). When a patient started the HCV therapy after the first LSM (LSM1) and achieved a sustained virological response (SVR), the follow-up was interrupted.

**Figure 1:**
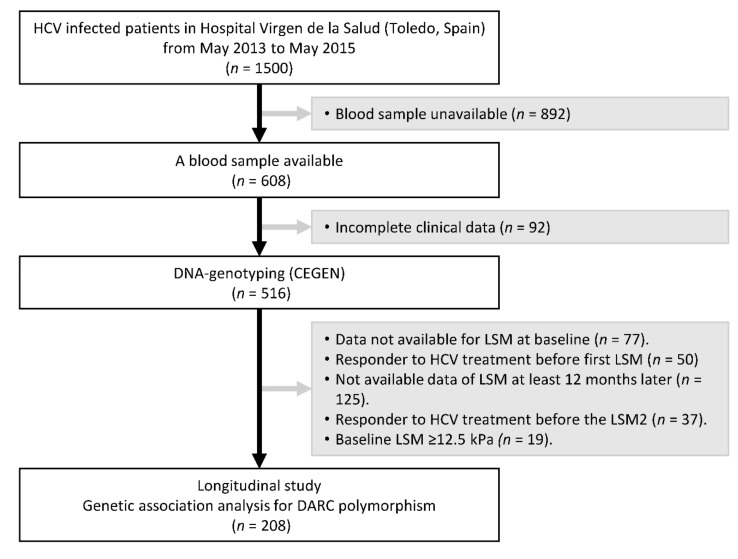
Flow diagram describing the inclusion and exclusion criteria of study participants. HCV: Hepatitis C virus; LSM: liver stiffness measurement; CEGEN: Spanish National Genotyping Center; DARC: Duffy antigen receptor for chemokines.

Figure 1 shows the flow diagram describing the inclusion and exclusion criteria of the study participants. About 1500 patients infected with HCV were attended at the Hospital Virgen de la Salud from May 2013 to May 2015. We had 608 blood samples available, but 92 patients’ samples were discarded due to incomplete clinical data. A total of 516 DNA samples were genotyped, but 308 were discarded for various reasons during the follow-up of the study: 77 patients did not have data of LSM, 50 patients were responders to HCV therapy before LSM1, 125 patients did not have data of LSM (LSM2) at least 12 months after baseline LSM (LSM1), 37 patients were responders to HCV therapy before 12 months of follow-up, and 19 patients had an LSM1 ≥ 12.5 kPa. A total of 208 patients fulfilled all selection criteria.

### 2.2. Clinical Data

The information of each patient was collected from medical records, as described previously [28], which included demographic, clinical, virological, and laboratory data. The clinic management of patients during the follow-up was performed according to clinical guidelines [29,30].

### 2.3. DNA Genotyping

Two hundred microliters of peripheral blood were used to extract total DNA with QIAsymphony DNA Mini Kit (Qiagen, Hilden, Germany). DNA genotyping was carried out at the CEGEN (Spanish National Genotyping Center; http://www.cegen.org/) using Agena Bioscience’s MassARRAY platform (San Diego, CA, USA) and the iPLEX^®^ Gold assay design system. As described by Gabriel et al. [31], the first assay consists of a locus-specific polymerase chain reaction (PCR) reaction, followed by single base extension using mass-modified dideoxynucleotide terminators of an oligonucleotide primer which anneals immediately upstream of the polymorphic site of interest. Using matrix-assisted laser desorption/ionization time-of-flight (MALDI-TOF) mass spectrometry, the distinct mass of the extended primer identifies the SNP allele. The quality control was carried out as follows: (i) technical replicates were performed by including duplicated samples on each plate; (ii) three positive Coriell controls (NA10860, NA10861, NA11984) from the Human Genetic Cell Repository were analyzed to ensure a technically correct process; (iii) a negative control was included to exclude DNA contamination; (iv) genotyping call rate over 95% was used.

Other SNPs analyzed in this population have been previously reported regarding their association with fibrosis progression: *PNPLA3* rs738409 [32], *MERTK* rs4374383 [33], and *IL7RA* rs6897932 [28].

### 2.4. Liver Stiffness Measurement

The LSM was evaluated by transient elastography (FibroScan^®^, Echosens, Paris, France) with a single machine and by a trained hepatologist as previously described by us [28]. Measurements were expressed in kilopascals (kPa) and considered reliable when the interquartile range-to-median ratio for at least ten successful measurements was lower than 0.30. Several cutoffs of LSM were used to stratify patients: <7.1 kPa (F0/F1—absence of or mild fibrosis), 7.1–9.4 kPa (F2—significant fibrosis), 9.5–12.4 kPa (F3—advanced fibrosis), and ≥12.5 kPa (F4—cirrhosis) [34].

### 2.5. Outcome Variable

The study period ran between the date of the first LSM (LSM1) and the date of the last LSM (LSM2), or the date of starting HCV therapy in responder patients who cleared HCV infection. The primary outcome was the change in values of LSM during the follow-up. We evaluated the change during the follow-up in LSM values as the ratio LSM2/LSM1 and the increase of LSM (ΔLSM = LSM2 − LSM1) through a simple summary statistical approach for repeated measurements [35,36]. This strategy condenses the repeated measurements to a single number per subject and provides valid results that are easily understood. Furthermore, the LSM2/LSM1 ratio provides the advantage of having no negative values, and thus a logarithmic transformation could normalize it. The ΔLSM was used for evaluating clinically relevant changes in LSM values: 5 kPa (ΔLSM ≥ 5 kPa), 7 kPa (ΔLSM ≥ 7 kPa), and 10 kPa (ΔLSM ≥ 10 kPa). The cirrhosis progression (F4; LSM ≥ 12.5 kPa) was measured as +1 (if F ≤ 3 (LSM < 12.5 kPa) changed to F4 (LSM ≥ 12.5 kPa)) or 0 (if F ≤ 3 did not change to F4).

### 2.6. Statistical Analysis

To compare independent groups, we used the Chi-squared test or Fisher’s exact test (categorical variables) and Mann–Whitney U-test (continuous variables). To compare repeated measurements, we used the Sign test (categorical variables) and the Wilcoxon signed-rank test (continuous variables).

We used generalized linear models (GLM) to analyze the genetic association between *DARC* rs12075 SNP and outcome variables. Firstly, a GLM with a gamma distribution (log-link) was used for analyzing continuous variables (LSM2/LSM1) and a GLM with a binomial distribution (logit-link) used for analyzing dichotomous variables (ΔLSM ≥ 5 kPa, ΔLSM ≥ 7 kPa, ΔLSM ≥ 10 kPa, and progression to cirrhosis). These tests provide the differences between groups, the arithmetic mean ratio (AMR), and the odds ratio (OR). The GLM tests were adjusted by the most relevant clinical and epidemiological variables by using the stepwise algorithm (at each step, covariables with a *p*-value < 0.20 are considered for entry). The covariables used were gender, age, time since HCV diagnosis, diabetes, high alcohol intake, HCV genotype, injection drug use, baseline LSM, HCV antiviral therapy before baseline, HCV antiviral therapy during the follow-up (patients who failed therapy), time of follow-up, and other SNPs previously reported in this study population (*PNPLA3* rs738409 [32], *MERTK* rs4374383 [33], and *IL7RA* rs6897932 [28]).

All statistical tests were performed with the Statistical Package for the Social Sciences (SPSS) 22.0 software (IBM Corp., Chicago, USA) and Stata 15.0 (StataCorp, Texas, USA). All *p*-values were two-tailed, and statistical significance was defined as *p* < 0.05.

## 3. Results

### 3.1. Characteristics of the Study Population

Table 1 describes the baseline characteristics of 208 patients infected by HCV without cirrhosis (LSM < 12.5 kPa). About 50% of the patients were men, the average age was 47 years old, less than 15% reported a high alcohol intake, and 10% were former injectable drug users. Regarding virological aspects, about 85% were infected with HCV genotype 1 and 22% had previously failed the HCV antiviral therapy (22 IFN-α, 1 IFN-α/RBV, 22 peg-IFN-α/RBV, and 2 DAAs + peg-IFN-α/RBV). Of these 208 patients, 88 had the rs12075 AA genotype, 92 had the rs12075 AG genotype, and 28 had the rs12075 GG genotype. No significant differences between the groups were found when patients were stratified by the rs12075 genotype. Moreover, the time intervals between the two selected LSM values (LSM1 and LSM2) were similar between the patients stratified by the rs12075 genotype (50.6 months (29.1; 64.2) in the AA genotype, 45.9 months (29.3; 60.5) in the AG genotype, and 38.4 months (26.8; 54.9) in the GG genotype; *p* = 0.400).

### 3.2. Characteristics of the DARC rs12075 SNP

The allelic and genotypic frequencies of the rs12075 SNP are shown in Table 2. The rs12075 SNP displayed <5% of missing values, had a minimum allele frequency of >30%, and was in Hardy–Weinberg equilibrium (*p* = 0.718). Furthermore, no significant differences were found in allelic and genotypic frequencies between HCV-infected patients and healthy subjects (an Iberian population in Spain (IBS)) from the 1000 Genomes Project Phase 3 (www.ensembl.org)).

### 3.3. DARC rs12075 SNP and Fibrosis Progression

The genetic association was evaluated according to additive, recessive, and dominant inheritance models, but we selected the dominant model because it was the one that best fitted our data.

**Figure 2:**
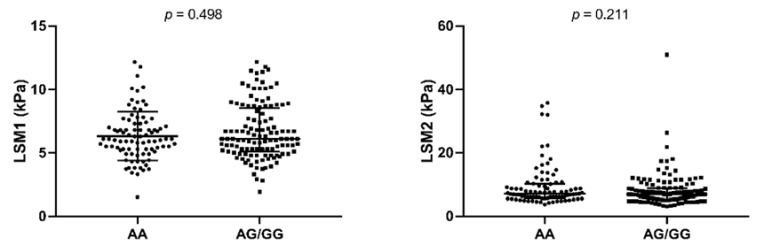
Summary of LSM values stratified by *DARC* rs12075 genotype (dominant model). *P*-values were calculated by Mann–Whitney test. LSM, liver stiffness measure; LSM1, baseline LSM; LSM2, final LSM.

Figure 2 shows the LSM values stratified by rs12075 genotype (dominant model). We did not find any significant differences in LSM values between AA and AA/AG genotypes when we analyzed baseline (LSM1) and the end of follow-up (LSM2) as two independent cross-sectional studies.

Table 3 shows the evolution of liver stiffness characteristics in HCV-infected patients during the follow-up. Overall, during a follow-up of 46.6 months, the percentage of patients with F0/F1 decreased (*p* < 0.001) and LSM values and the percentage of patients with cirrhosis increased (*p* < 0.001). This pattern of changes was maintained in all groups of patients analyzed according to their rs12075 genotype (AA and AG/GG). However, the variations in liver stiffness characteristics were lower in patients with the rs12075 AG/GG genotype (AG/GG versus AA) (Table 4). Thereby, in the adjusted analysis, patients with the rs12075 AG/GG genotype had a lower risk of having increased values of LSM2/LSM1 ratio (AMR = 0.83; *p* = 0.001) and of showing an increase in LSM ≥ 5 kPa (OR = 0.28; *p* = 0.009). Besides, patients with rs12075 AG/GG had a lower risk of cirrhosis progression (OR = 0.24; *p* = 0.009). No significant values were found for an increase in LSM ≥ 10 kPa (Table 4). 

## 4. Discussion

The finding of genetic markers that predict the evolution of liver fibrosis in HCV-infected patients would be very valuable to initiate preventive measures aimed at avoiding liver deterioration. In this retrospective study, noncirrhotic patients with chronic hepatitis C infection were evaluated for liver stiffness twice with an interval of at least 12 months. Although the percentage of patients with cirrhosis increased in the analyzed *DARC* rs12075 genotypes (AG/GG versus AA) during the study, the variations in liver stiffness were lower in patients with the rs12075 AG/GG genotype, resulting in lower risk of cirrhosis progression.

DARC has an important role in leukocyte recruitment and inflammatory chemokine homeostasis, and some evidence indicates that it attenuates the inflammatory response, probably by mediating chemokine binding and internalization in red blood cells, and thus reducing the levels of free chemokines [21,22,23,24,25,37,38]. Internalized chemokines are, however, not degraded and they can be released, in some conditions, to promote local inflammation.

The *DARC* rs12075 polymorphism (Asp42Gly) is a nonsynonymous SNP that forms the basis for Duffy blood groups Fy^b^ (Asp, A allele) and Fy^a^ (Gly, G allele). The rs12075 SNP is related to approximately 20% of the variation in serum CCL2 (MCP-1) concentration and has also been associated with serum IL8 and RANTES concentrations [26], but a full explanation for this relationship has not been found. Schnabel et al. suggested that the highest levels of serum CCL2 in rs12075 AA homozygote subjects may be due to a genotype-mediated change in DARC function related to necessary sulfation at Tyr41 rather than a change in expression [26]. Thus, more efficient sulfation of Tyr41 in the presence of negatively charged Asp may promote more efficient chemokine binding to erythrocytes. These chemokines captured and internalized by the erythrocytes would be released during the blood clotting process. In agreement with this hypothesis, several articles have shown a decrease in the levels of CCL2 and CXCL8 (IL-8) in serum from persons with the rs12075 G allele [26,39,40,41]. Besides, the rs12075 G allele has also been related with low CCL2 levels in patients with chronic inflammatory diseases such as ischemia of the lower extremities [42] and obesity [43], as well as low leukocyte counts in African people [44].

The monocyte chemoattractant protein-1 (CCL2) is involved in the recruitment of leukocytes to sites of inflammation and it is a profibrotic chemokine upregulated during liver fibrogenesis [45]. Therefore, it is not surprising that, as found in the present study, the *DARC* rs12075 polymorphism had an important impact on CHC progression. Advanced liver fibrosis and cirrhosis in HCV infections are associated with increased thrombotic risks [46]. In this scenario, levels of chemokines released by erythrocytes in the liver due to coagulation processes would be higher in individuals with the *DARC* rs12075 AA genotype, which would explain their higher risk of liver fibrosis progression. In the only previous study that we found, rs12075 SNP was not associated with the severity of liver disease in patients infected with HCV [47]. However, as pointed out by the authors, a limiting factor of their study was its cross-sectional design. Since liver fibrosis is a dynamic process, a longitudinal study, such as the one reported here, is a better approach to understand this process.

### Limitations of the Study

Firstly, this is a retrospective study, which may have ascertainment and selection biases, but the design of repeated measures provides robustness to our study (each patient served as his or her control). Secondly, this study had a limited sample size, which may impair the ability to detect stronger associations, such as the association between *DARC* rs12075 SNP and the increase in LSM greater than 10 kPa. Thirdly, the time between LSM1 and LSM2 (follow-up time) was different among subjects, but all patients had more than 12 months of follow-up and 75% of the patients had more than 28 months of follow-up. In addition, the time of follow-up was similar in patients stratified by rs12075 genotypes. Fourthly, we did not have access to some crucial clinical variables (such as metabolic syndrome, obesity, abdominal ultrasound, and liver pathological study, among others) and biomarkers (such as platelet counts, transaminases, HCV viral load, AST to Platelet Ratio Index (APRI) score, and Fibrosis-4 (FIB-4) index, among others) due to the retrospective design of our study. We first selected the LSM data (the first available, corresponding to a specific date) and then we collected the clinical data from clinical records. In this way, many patients did not have available data for some variables at specific time points when the LSM was measured. In addition, we did not have plasma samples available, so the plasma concentrations of chemokines (IP-10, MCP-1, and IL-8) and cytokines (IL-12, IL-15, IFN-gamma, and IL-10) were missing. Fifthly, more than 20% of the patients were nonresponders to previous interferon therapy, but they were included in the study because this HCV therapy does not appear to protect against CHC progression in the long term [48].

## 5. Conclusions

In summary, we found an association between *DARC* rs12075 SNP and CHC progression. Specifically, patients with the *DARC* rs12075 AG/GG genotype had a lower risk of liver fibrosis progression and development of cirrhosis. Given the role of DARC in inflammation and leukocyte trafficking, further analyses of *DARC* SNPs may lead to a better understanding of the immune pathogenesis of chronic hepatitis C, as well as the early identification of high-risk patients.

## Figures and Tables

**Table 1:** Clinical and epidemiological characteristics of hepatitis C virus (HCV)-infected patients at baseline.

		*DARC* rs12075 Polymorphism			
Characteristic	All Patients	AA	AG	GG	*p*-Value
No.	208	88	92	28	
Male	112 (53.8%)	44 (50%)	56 (60.9%)	12 (4.9%)	0.156
Age (years)	47.1(41.5; 57.6)	49.2 (40.7; 56.1)	46.5 (42.8; 55.2)	48.8 (42.4; 59.6)	0.830
Time of HCV infection (years)	8.2 (3.2; 13.2)	9.3 (3.4; 13.6)	8.05 (3.6; 12.9)	4.22 (1.1; 11.0)	0.694
High alcohol intake	28 (13.5%)	11 (12.5%)	14 (15.2%)	3 (10.7%)	0.781
Prior injection drug use	21 (10.1%)	9 (10.2%)	11 (12%)	1 (3.6%)	0.517
HCV genotype (*n* = 204)					
1	174 (85.3%)	75 (88.2%)	78 (84.8%)	21 (77.8%)	0.402
3	14 (6.9%)	6 (7.1%)	5 (5.4%)	3 (11.1%)	0.588
4	15 (7.4%)	4 (4.7%)	8 (8.7%)	3 (11.1%)	0.432
5	1 (0.5%)	0 (0%)	1 (1.1%)	-	-
Prior failed HCV therapy	47 (22.6%)	20 (22.7%)	22 (23.9%)	5 (17.9%)	0.798
IFN-α	22 (10.6%)	10 (11.4%)	9 (9.8%)	3 (10.7%)	0.942
IFN-α/RBV	1 (0.5%)	-	1 (1.1%)	-	-
peg-IFN-α/RBV	22 (10.6%)	10 (11.4%)	10 (10.9%)	2 (7.1%)	0.812
DAAs + peg-IFN-α/RBV	2 (1%)	-	2 (2.2%)	-	-
Baseline LSM (kPa)	6.1 (5.2; 7.7)	6.1 (5.3; 7.2)	6.1 (5.3; 8.7)	5.6 (4.9; 7.4)	0.280
F0–F1 (<7.1 kPa)	149 (71.6%)	66 (75%)	62 (67.4%)	21 (75.0%)	0.481
F2 (7.1–9.4 kPa)	38 (18.3%)	16 (18.2%)	19 (20.7%)	3 (10.7%)	0.432
F3 (9.5–12.4 kPa)	21 (10.1%)	6 (6.8%)	11 (12.0%)	4 (14.3%)	0.315

**Statistics:** Values expressed as absolute numbers (%) or median (25th percentile; 75th percentile). *p*-values were estimated with the nonparametric Mann–Whitney U-test for continuous variables or Chi-squared test for categorical variables. HCV, hepatitis C virus; LSM, liver stiffness measurement; kPa, kilopascal; DAAs, direct-acting antivirals; peg-IFN-α/RBV, peg-interferon-alpha/ribavirin; DARC, Duffy antigen/chemokine receptor; F0–F1, absence of or mild fibrosis; F2, significant fibrosis; F3, advanced fibrosis.

**Table 2:** Summary of Hardy–Weinberg equilibrium test and allelic and genotypic frequencies for *DARC* rs12075 polymorphism in HCV-infected patients compared to an Iberian population (data from 1000 Genomes Project) (www.ensembl.org).

		HCV Cohort	IBS Group	*p*-Value
No.		208	107	
Alleles	A	64.4%	70.1%	0.312
	G	35.6%	29.9%	
Genotype	AA	42.3%	49.5%	0.160
	AG	44.2%	41.1%	
	GG	13.5%	9.3%	
HWE (*p*-value)		0.718	0.856	

**Statistics:***p*-values were calculated by Chi-squared test. HWE, Hardy–Weinberg equilibrium; IBS, Iberian population in Spain.

**Table 3:** Summary of clinical characteristics related to liver fibrosis in patients infected with HCV during the follow-up.

	All Patients			rs12075 AA Genotype			rs12075 AG/GG Genotype		
Characteristic	Baseline	End	*p*-Value	Baseline	End	*p* -Value	Baseline	End	*p* -Value
Follow-up (months)	46.6 (28.7; 61.5)			50.6 (29.1; 64.2)			43.8 (28.2; 60.2)		
LSM (kPa)	6.1 (5.2; 7.7)	6.8 (5.5; 9.4)	<0.001	6.1 (5.3; 7.2)	7.1 (5.8; 10.1)	<0.001	6.1 (5.1; 8.5)	6.8 (5.3; 8.8)	<0.001
F0–F1 (<7.1 kPa)	149 (71.6%)	110 (52.9%)	<0.001	66 (75%)	44 (50%)	<0.001	83 (69.2%)	66 (55%)	0.004
F2 (7.1–9.4 kPa)	38 (18.3%)	47 (22.6%)	0.382	16 (18.2%)	21 (23.9%)	0.584	22 (18.3%)	26 (21.7%)	0.607
F3 (9.5–12.4 kPa)	21 (10.1%)	25 (12%)	0.055	6 (6.8%)	8 (9.1%)	0.077	15 (12.5%)	17 (14.2%)	0.361
F4 (≥12.5 kPa)	0 (0%)	26 (12.5%)	<0.001	0 (0%)	15 (17%)	<0.001	0 (0%)	11 (9.2%)	0.001

**Statistics:** Values expressed as absolute numbers (%) or median (25th percentile; 75th percentile). *p*-values were estimated with the nonparametric Wilcoxon test for continuous variables or Sign test for categorical variables. LSM, liver stiffness measurement; kPa, kilopascal; peg-IFN-α/RBV, peg-interferon-alpha/ribavirin;

**Table 4:** Relationship between *DARC* rs12075 polymorphism and liver fibrosis progression in HCV-infected patients (longitudinal study).

	*DARC* rs12075 Genotypes		Unadjusted		Adjusted	
Outcome	AA	AG/GG	Exp(B) (95% CI)	*p* Value ^a^	Exp(B) (95% CI)	*p* Value ^b^
LSM2/LSM1	1.26 (0.98; 1.51)	1.10 (0.94; 1.31)	0.79 (0.71; 0.87)	<0.001	0.83 (0.75; 0.93)	0.001
ΔLSM ≥ 5 kPa	16 (18.2%)	9 (7.5%)	0.36 (1.53; 0.87)	0.023	0.28 (0.10; 0.73)	0.009
ΔLSM ≥ 7 kPa	10 (11.4%)	4 (3.3%)	0.27 (0.08; 0.88)	0.031	0.31 (0.07; 1.27)	0.104
ΔLSM ≥ 10 kPa	7 (8%)	4 (3.3%)	0.39 (0.11; 1.41)	0.153	0.47 (0.11; 2.06)	0.322
Progression to F4	15 (17%)	11 (9.2%)	0.49 (0.21; 1.13)	0.094	0.24 (0.08; 0.70)	0.009

**Statistics:**^a^, *p*-values were calculated by univariate regression; ^b^, *p*-values were calculated by multivariate regression adjusted by the most important clinical and epidemiological characteristics (see Section 2.6). Statistically significant differences are shown in bold. Exp(B), exponentiation of the B coefficient, which was an arithmetic mean ratio (AMR) for continuous variables and an odds ratio (OR) for categorical variables; 95% CI, 95% of confidence interval; F4, cirrhosis; DARC, Duffy antigen/chemokine receptor.

## References

[1] WHO (2017). Global Hepatitis Report.

[2] Lagging L.M., Westin J., Svensson E., Aires N., Dhillon A.P., Lindh M., Wejstal R., Norkrans G. (2002). Progression of fibrosis in untreated patients with hepatitis C virus infection. Liver.

[3] Hoofnagle J.H. (2002). Course and outcome of hepatitis C. Hepatology.

[4] Conti F., Buonfiglioli F., Scuteri A., Crespi C., Bolondi L., Caraceni P., Foschi F.G., Lenzi M., Mazzella G., Verucchi G. (2016). Early occurrence and recurrence of hepatocellular carcinoma in HCV-related cirrhosis treated with direct-acting antivirals. J. Hepatol..

[5] Forner A., Reig M., Bruix J. (2018). Hepatocellular carcinoma. Lancet.

[6] Rueger S., Bochud P.Y., Dufour J.F., Mullhaupt B., Semela D., Heim M.H., Moradpour D., Cerny A., Malinverni R., Booth D.R. (2015). Impact of common risk factors of fibrosis progression in chronic hepatitis C. Gut.

[7] Heim M.H., Bochud P.Y., George J. (2016). Host—Hepatitis C viral interactions: The role of genetics. J. Hepatol..

[8] Pawlotsky J.M., Aghemo A., Back D., Dusheiko G., Forns X., Puoti M., Sarrazin C. (2015). EASL recommendations on treatment of hepatitis C 2015. J. Hepatol..

[9] Thiele M., Kjaergaard M., Thielsen P., Krag A. (2017). Contemporary use of elastography in liver fibrosis and portal hypertension. Clin. Physiol. Funct. Imaging.

[10] Tsochatzis E.A., Bosch J., Burroughs A.K. (2014). Liver cirrhosis. Lancet.

[11] Resino S., Sanchez-Conde M., Berenguer J. (2012). Coinfection by human immunodeficiency virus and hepatitis C virus: Noninvasive assessment and staging of fibrosis. Curr. Opin. Infect. Dis..

[12] Castera L. (2015). Noninvasive assessment of liver fibrosis. Dig. Dis..

[13] Zeremski M., Dimova R., Brown Q., Jacobson I.M., Markatou M., Talal A.H. (2009). Peripheral CXCR3-associated chemokines as biomarkers of fibrosis in chronic hepatitis C virus infection. J. Infect. Dis..

[14] Fallahi P., Ferrari S.M., Giuggioli D., Sebastiani M., Colaci M., Ferri C., Antonelli A. (2017). Chemokines in the pathogenesis and as therapeutical markers and targets of HCV chronic infection and HCV extrahepatic manifestations. Curr. Drug Targets.

[15] Promrat K., McDermott D.H., Gonzalez C.M., Kleiner D.E., Koziol D.E., Lessie M., Merrell M., Soza A., Heller T., Ghany M. (2003). Associations of chemokine system polymorphisms with clinical outcomes and treatment responses of chronic hepatitis C. Gastroenterology.

[16] Hellier S., Frodsham A.J., Hennig B.J., Klenerman P., Knapp S., Ramaley P., Satsangi J., Wright M., Zhang L., Thomas H.C. (2003). Association of genetic variants of the chemokine receptor CCR5 and its ligands, rantes and MCP-2, with outcome of HCV infection. Hepatology.

[17] Mantovani A., Locati M., Vecchi A., Sozzani S., Allavena P. (2001). Decoy receptors: A strategy to regulate inflammatory cytokines and chemokines. Trends Immunol..

[18] Marsh W.L. (1975). Present status of the duffy blood group system. CRC Crit. Rev. Clin. Lab. Sci..

[19] Gardner L., Patterson A.M., Ashton B.A., Stone M.A., Middleton J. (2004). The human duffy antigen binds selected inflammatory but not homeostatic chemokines. Biochem. Biophys. Res. Commun..

[20] Chaudhuri A., Nielsen S., Elkjaer M.L., Zbrzezna V., Fang F., Pogo A.O. (1997). Detection of Duffy antigen in the plasma membranes and caveolae of vascular endothelial and epithelial cells of nonerythroid organs. Blood.

[21] Pruenster M., Mudde L., Bombosi P., Dimitrova S., Zsak M., Middleton J., Richmond A., Graham G.J., Segerer S., Nibbs R.J. (2009). The Duffy antigen receptor for chemokines transports chemokines and supports their promigratory activity. Nat. Immunol..

[22] Lee J.S., Frevert C.W., Wurfel M.M., Peiper S.C., Wong V.A., Ballman K.K., Ruzinski J.T., Rhim J.S., Martin T.R., Goodman R.B. (2003). Duffy antigen facilitates movement of chemokine across the endothelium in vitro and promotes neutrophil transmigration in vitro and in vivo. J. Immunol..

[23] Darbonne W.C., Rice G.C., Mohler M.A., Apple T., Hebert C.A., Valente A.J., Baker J.B. (1991). Red blood cells are a sink for interleukin 8, a leukocyte chemotaxin. J. Clin. Investig..

[24] Yamamoto A., Saito N., Ogasawara S., Shiratori T., Kondo J., Itoga M., Kayaba H. (2017). Intracellular storage of duffy antigen-binding chemokines by duffy-positive red blood cells. Clin. Lab..

[25] Fukuma N., Akimitsu N., Hamamoto H., Kusuhara H., Sugiyama Y., Sekimizu K. (2003). A role of the duffy antigen for the maintenance of plasma chemokine concentrations. Biochem. Biophys. Res. Commun..

[26] Schnabel R.B., Baumert J., Barbalic M., Dupuis J., Ellinor P.T., Durda P., Dehghan A., Bis J.C., Illig T., Morrison A.C. (2010). Duffy antigen receptor for chemokines (*DARC*) polymorphism regulates circulating concentrations of monocyte chemoattractant protein-1 and other inflammatory mediators. Blood.

[27] Marra F., Tacke F. (2014). Roles for chemokines in liver disease. Gastroenterology.

[28] Jimenez-Sousa M.A., Gomez-Moreno A.Z., Pineda-Tenor D., Medrano L.M., Sanchez-Ruano J.J., Fernandez-Rodriguez A., Artaza-Varasa T., Saura-Montalban J., Vazquez-Moron S., Ryan P. (2018). The *IL7RA* rs6897932 polymorphism is associated with progression of liver fibrosis in patients with chronic hepatitis C: Repeated measurements design. PLoS ONE.

[29] Calvaruso V., Craxì A. (2012). 2011 european association of the study of the liver hepatitis C virus clinical practice guidelines. Liver Int..

[30] European Association for Study of Liver (2014). EASL clinical practice guidelines: Management of hepatitis C virus infection. J. Hepatol..

[31] Gabriel S., Ziaugra L., Tabbaa D., Haines J.L. (2009). SNP genotyping using the sequenom MassARRAY iPLEX platform. Current Protocols in Human Genetics.

[32] Jimenez-Sousa M.A., Gomez-Moreno A.Z., Pineda-Tenor D., Sanchez-Ruano J.J., Fernandez-Rodriguez A., Artaza-Varasa T., Gomez-Sanz A., Martin-Vicente M., Vazquez-Moron S., Resino S. (2018). *PNPLA3* rs738409 polymorphism is associated with liver fibrosis progression in patients with chronic hepatitis C: A repeated measures study. J. Clin. Virol..

[33] Jimenez-Sousa M.A., Gomez-Moreno A.Z., Pineda-Tenor D., Brochado-Kith O., Sanchez-Ruano J.J., Artaza-Varasa T., Gomez-Sanz A., Fernandez-Rodriguez A., Resino S. (2018). The myeloid-epithelial-reproductive tyrosine kinase (*MERKT*) rs4374383 polymorphism predicts progression of liver fibrosis in hepatitis C virus-infected patients: A longitudinal study. J. Clin. Med..

[34] Castera L., Vergniol J., Foucher J., Le Bail B., Chanteloup E., Haaser M., Darriet M., Couzigou P., De Ledinghen V. (2005). Prospective comparison of transient elastography, fibrotest, APRI, and liver biopsy for the assessment of fibrosis in chronic hepatitis C. Gastroenterology.

[35] Albert P.S. (1999). Longitudinal data analysis (repeated measures) in clinical trials. Stat. Med..

[36] Senn S., Stevens L., Chaturvedi N. (2000). Repeated measures in clinical trials: Simple strategies for analysis using summary measures. Stat. Med..

[37] Mangalmurti N.S., Xiong Z., Hulver M., Ranganathan M., Liu X.H., Oriss T., Fitzpatrick M., Rubin M., Triulzi D., Choi A. (2009). Loss of red cell chemokine scavenging promotes transfusion-related lung inflammation. Blood.

[38] Akalin E., Neylan J.F. (2003). The influence of duffy blood group on renal allograft outcome in African Americans. Transplantation.

[39] Aragones G., Ercilla A., Barreda M., Rull A., Beltran-Debon R., Rodriguez-Gallego E., Alonso-Villaverde C., Camps J., Joven J. (2012). Human duffy blood group alloantigen system influences the measurement of monocyte chemoattractant protein-1 (MCP-1) in serum but not in plasma. Clin. Lab..

[40] Naitza S., Porcu E., Steri M., Taub D.D., Mulas A., Xiao X., Strait J., Dei M., Lai S., Busonero F. (2012). A genome-wide association scan on the levels of markers of inflammation in Sardinians reveals associations that underpin its complex regulation. PLoS Genet..

[41] Moreno Velasquez I., Kumar J., Bjorkbacka H., Nilsson J., Silveira A., Leander K., Berglund A., Strawbridge R.J., Arnlov J., Melander O. (2015). Duffy antigen receptor genetic variant and the association with interleukin 8 levels. Cytokine.

[42] Rull A., Hernandez-Aguilera A., Fibla M., Sepulveda J., Rodriguez-Gallego E., Riera-Borrull M., Sirvent J.J., Martin-Paredero V., Menendez J.A., Camps J. (2014). Understanding the role of circulating chemokine (C-C motif) ligand 2 in patients with chronic ischemia threatening the lower extremities. Vasc. Med..

[43] Voruganti V.S., Laston S., Haack K., Mehta N.R., Smith C.W., Cole S.A., Butte N.F., Comuzzie A.G. (2012). Genome-wide association replicates the association of duffy antigen receptor for chemokines (*DARC*) polymorphisms with serum monocyte chemoattractant protein-1 (MCP-1) levels in Hispanic children. Cytokine.

[44] Crosslin D.R., McDavid A., Weston N., Nelson S.C., Zheng X., Hart E., de Andrade M., Kullo I.J., McCarty C.A., Doheny K.F. (2012). Genetic variants associated with the white blood cell count in 13,923 subjects in the emerge network. Hum. Genet..

[45] Marra F., DeFranco R., Grappone C., Milani S., Pastacaldi S., Pinzani M., Romanelli R.G., Laffi G., Gentilini P. (1998). Increased expression of monocyte chemotactic protein-1 during active hepatic fibrogenesis: Correlation with monocyte infiltration. Am. J. Pathol..

[46] Gonzalez-Reimers E., Quintero-Platt G., Martin-Gonzalez C., Perez-Hernandez O., Romero-Acevedo L., Santolaria-Fernandez F. (2016). Thrombin activation and liver inflammation in advanced hepatitis C virus infection. World J. Gastroenterol..

[47] Lettow I., Berres M.L., Schmitz P., Muller T., Berg T., Neumann U.P., Trautwein C., Wasmuth H.E. (2011). A duffy antigen receptor for chemokines (*DARC*) polymorphism that determines pro-fibrotic chemokine serum concentrations is not directly associated with severity of hepatitis C infection. Hum. Immunol..

[48] Carmona I., Cordero P., Ampuero J., Rojas A., Romero-Gomez M. (2016). Role of assessing liver fibrosis in management of chronic hepatitis C virus infection. Clin. Microbiol. Infect..

